# Myogenic Differentiation of Stem Cells for Skeletal Muscle Regeneration

**DOI:** 10.1155/2021/8884283

**Published:** 2021-02-04

**Authors:** Dengjie Yu, Zijun Cai, Daishi Li, Yi Zhang, Miao He, Yuntao Yang, Di Liu, Wenqing Xie, Yusheng Li, Wenfeng Xiao

**Affiliations:** ^1^Department of Orthopedics, Xiangya Hospital, Central South University, Changsha, 410008 Hunan, China; ^2^National Clinical Research Center for Geriatric Disorders, Xiangya Hospital, Central South University, Changsha, 410008 Hunan, China; ^3^Department of Dermatology, Xiangya Hospital, Central South University, Changsha, Hunan, China

## Abstract

Stem cells have become a hot research topic in the field of regenerative medicine due to their self-renewal and differentiation capabilities. Skeletal muscle tissue is one of the most important tissues in the human body, and it is difficult to recover when severely damaged. However, conventional treatment methods can cause great pain to patients. Stem cell-based tissue engineering can repair skeletal muscle to the greatest extent with little damage. Therefore, the application of stem cells to skeletal muscle regeneration is very promising. In this review, we discuss scaffolds and stem cells for skeletal muscle regeneration and put forward our ideas for future development.

## 1. Introduction

Skeletal muscle accounts for 30-40% of the weight of a healthy human body and is necessary for free movement of the human body [1]. Muscle regeneration relies on a group of small adult stem cells called satellite cells. Satellite cells are quiescent under resting conditions, but they can quickly reenter the cell cycle after being injured or receiving growth signals. Activated satellite cells will migrate and proliferate extensively for muscle regeneration [2]. Though healthy skeletal muscle has promising recovery ability to cope with minor injuries in daily life, the fate of satellite cells is strictly controlled by internal and external factors. This fragile balance may be disturbed by aging, hereditary myopathy, and massive muscle loss. Surgery is commonly used in clinical practice, but the high cost and considerable may discourage some patients. Under this circumstance, tissue engineering (TE) attracts the attention of researchers and has become the new tool to treat skeletal muscle diseases and promote skeletal muscle regeneration [3–5].

The three pillars of tissue engineering are cells, biomaterials, and environment [6]. Stem cells are undifferentiated cells that are presented in all parts of the body, which possesses the ability of self-renewal and differentiation. Since a number of stem cells have been proven to have the potential for myogenic differentiation, stem cells are considered the most potential cell source of skeletal muscle TE [7–9]. As one of the three elements of tissue engineering, scaffolds play an important role in the whole technology. The function of scaffolds is to simulate extracellular matrix. Because different tissues have their unique extracellular matrix, different types of scaffolds can steer cell differentiation towards different directions.

In this review, we describe the directional differentiation of skeletal muscle cells according to the sequence of three pillars of tissue engineering. We first introduced the process of skeletal muscle regeneration under physiological conditions and the chemical signals expressed at each differentiation stage. Second, we introduced some biomaterials and environmental factors currently used for myogenic differentiation. Third, we listed several commonly used stem cells for myogenic differentiation and described the recent advances in directing into skeletal muscle. Finally, we put forward our own views on myogenic differentiation and make an outlook on its future use.

## 2. Muscle Regeneration

### 2.1. Myogenic Markers

**Figure 1:**
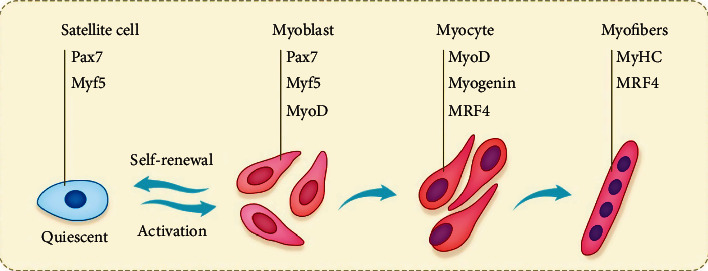
Schematic diagram of muscle regeneration. Skeletal muscle tissue regeneration is regulated by a genetic cascade involving Pax7 and MRFs, which drive every step of satellite cell activation, transient expansion of progenitor cells, and the differentiation and formation of new muscle fibers. Interestingly, satellite cell self-renewal can retain a small number of rested cells after regeneration to meet future regeneration needs.

In adult muscles, satellite cells are usually mitotically quiescent. In general, once exposed to signals from the damaged environment, satellite cells will leave their quiescent state, reenter the cell cycle, and start proliferating (satellite cell activation). Some daughter cells continue to differentiate, while others return to quiescence to replenish the reserve population of satellite cells, then, the activated satellite cells form multinucleated myotubes after mitosis [10, 11]. Many pathological conditions, such as muscular dystrophies (MDs) or muscle wasting, cannot provide sufficient signals for satellite cells, which will impair their regeneration potential [2]. The multistep muscle formation process is strictly controlled by a complex gene regulatory network [12]. First, many miRNAs (microRNAs) are small noncoding RNA molecules that target mRNA, are used to fine-tune gene expression, and are also an important part of the network [13, 14]. Second, muscle satellite cells can be characterized by a combination of several genetic markers, including paired box proteins, Pax7 (considered a clear MUSCs marker), and muscle regulatory factors MRFs, including MYF5, MYOD, MYOG (myogenin), and MRF4 [15–17]. PAX7 is a paired homeobox transcription factor, which specifies the myogenic properties of muscle stem cells and acts as a nodal factor by stimulating proliferation and inhibiting differentiation [18]. Some studies have shown that after Pax7 is missing, satellite cells and myoblasts show cell cycle arrest and imbalance of myogenic regulatory factors. In a word, Pax7 is an absolute requirement for the function of adult skeletal muscle satellite cells [19]. Third, myogenesis depends on the precise and dynamic integration of multiple Wnt signals, this allows the self-renewal and progress transcription factors such as myogenic factor 5 (MYF5), and myogenic differentiation factor 1 (MYOD) can be specifically expressed in myogenic cells, but not expressed in stationary satellite cells [2]. On the one hand, MYOD is a key transcription factor for myogenesis. On the other hand, the inactivation of MRF4 will cause subtle changes in muscle strength and innervation [20]. Researchers have shown that adult satellite cells originated from progenitor cells that first expressed the myogenic assay gene MYF5 in the fetal stage of myogenesis [21] (see Figure 1). In addition, during embryonic development, Wnt signals control the expression of myogenic regulatory factors (MRFs), and MRFs are essential for the development of myogenic lineages [22]. Above all, the formation of skeletal muscle is a process strictly regulated of muscle precursors in the myogenic lineage [23]. It is worth noting that the resting state of satellite cells has some similarities with hibernation, in which the cells are kept in a minimum energy state. The energy needed comes from the catabolism of storing large molecules and can minimize energy consumption, thereby keeping cells at the forefront of cell and developmental biology [24].

### 2.2. MRF in Differentiation Stages

Muscle regeneration can be divided into several stages, which are characterized by different expression of myogenic regulatory factors (MRFs). In the stationary phase, satellite cells are not active, but ready to activate. In a way, quiescent satellite cells usually express markers such as Pax7 and Myf5. After muscle injury, satellite cells are stimulated by various signals generated by the injury environment and finally differentiate satellite cells and migrate to the injured site, then reenter the cell cycle to proliferate. At this stage, they are called myoblasts and express the myoblast marker Pax7, MYF5, and/or MYOD. After the proliferation phase, myoblasts exit the cell cycle and differentiate into mature muscle cells [2, 20, 21]. About 80% of Pax7+ cells express MYF5, but after activation and proliferation, the expression of Pax7 and MYF5 decreases, while MYOD increases correspondingly in the proliferative phase [20, 21, 25].

### 2.3. Advanced Studies Involved Muscle Regeneration

Researchers extracted extracellular matrix (ECM) from the thigh muscles of adult rats and presented it to the cells as a surface coating. They deserved that compared with standard growth noodles, myogenic cells cultured on ECM extracts have stronger proliferation and differentiation capabilities. It is confirmed that ECM molecules extracted from skeletal muscle can positively affect the proliferation and differentiation of satellite cells and myoblasts [26]. Rayagiri et al. found that skeletal satellite cells induced local remodeling of ECM and the deposition of laminin-*α*1 and laminin *α*5 into the basal layer of the satellite cell niche. Genetic ablation of laminin-*α*1, destruction of integrin-*α*6 signal, or destruction of matrix metalloproteinase activity can impair the expansion and self-renewal of satellite cells; it is proved that the remodeling of ECM is an essential process for stem cell activity to support reproduction and self-renewal [27]. Another researcher has proved that the presence of adipose tissue-derived stromal cells (ADSCs) derived from adipose tissue can promote skeletal muscle regeneration, and this effect can be enhanced by pretreatment of IL-4 and SDF-1 cells [28]. On the other hand, mesenchymal progenitors (MPs) are also involved in regeneration. Scott et al. determined that methylation in cancer 1 (Hic1) is a marker of skeletal muscle MP, and it further shows that the loss of Hic1 leads to the proliferation of MP. These suggest that Hic1 + MP coordinates many aspects of skeletal muscle regeneration by providing stage-specific immune regulation and nutritional and mechanical support. They further show that they have unique functions and genealogical potential. It can be concluded that HIC1 regulates MP quiescence and identifies MP subgroups with short-term and long-lasting effects in muscle regeneration [29].

### 2.4. Scaffolds in Tissue Engineering

#### 2.4.1. Cell Culture: Transition from 2D to 3D

**Figure 2:**
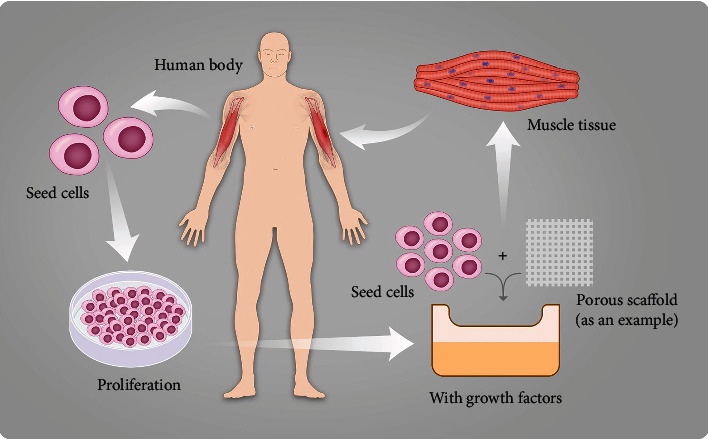
Schematic diagram of the general process of skeletal muscle tissue engineering. Taking the method of treating the biceps brachii defect with porous scaffolds as an example. First, seed cells are obtained from the biceps brachii on the healthy arm and are cultured in vitro. Next, we should make seed cells attached to porous scaffolds and add growth factors. Finally, a small amount of healthy skeletal muscle tissue is obtained and then implanted into the human body.

The method of two-dimensional (2D) cell culture is the basic method of cell culture. It first appeared in the early 20th century [30], which has existed for many years as the most extensive and conventional culture method of cells and plays an important role in stem cell research, biomedical fields, and so forth [31, 32]. However, this classical method was born with obvious imperfections [33, 34], because all of the cells in the human body are in a complex three-dimensional environment, and the cells cultured in 2D mode lack interaction with adjacent cells and extracellular matrix, resulting in cell signal imbalance and cell morphological changes [35]. In recent years, three-dimensional (3D) culture technology has gradually become one of the hot research fields in cell biology and tissue engineering (see Figure 2). The three elements of tissue engineering are seed cells, scaffolds, and growth factors [36, 37]. The cells cultured in 3D showed different characteristics from those in 2D. Therefore, it is in the foreseeable future that 2D cell culture gradually withdrew from the stage of history and was replaced by more perfect 3D cell culture technology. 3D cell culture technology has obvious advantages, but it will be a long process to completely replace 2D cell culture technology because 3D cell culture absolutely requires more funds, complex operation, and experience.

#### 2.4.2. A Brief Introduction of Scaffolds and Their Application Examples for Muscle Regeneration

The utilization of scaffolds is an indispensable part of tissue engineering, a useful technique for muscle regeneration, which can provide temporary mechanical support and necessary growth environment for seed cell adhesion, growth, proliferation, and differentiation [37]. Scaffolds are defined as three-dimensional (3D) solid biomaterials that play an indispensable role in tissue regeneration [35, 38]. The physical and chemical properties of scaffolds play an important role in three-dimensional cell culture, which always determines the fate of cells or the outcome of implantation. It is necessary to control these properties for various tissue engineering applications. According to the source, scaffold materials can be divided into natural materials, synthetic materials, and composite materials. The function of scaffolds in tissue engineering is to mimic the function of ECM [5]. ECM is unique in specific tissue whose properties are required for 3D scaffolds in engineering different tissue [39]. We should take many aspects into consideration when selecting scaffold: architecture, cell and tissue compatibility, and bioactivity and mechanical properties. Four main scaffold methods for tissue engineering have been developed rapidly including: premade porous scaffolds for cell seeding, 、decellularized ECM, cell sheets with self-secreted ECM, and cell encapsulation in self-assembled hydrogel matrix [36].

The past few decades have witnessed the development of applying tissue engineering techniques to muscle regeneration. Scaffolds used to support skeletal muscle regeneration should accommodate and promote the formation of densely packed, highly-aligned myofibers throughout a large tissue volume [5]. Scaffolds used for muscle regeneration should carry polarity-oriented property to maintain the parallel differentiation and growth of multinucleated myotubes. In addition, tension and elasticity are required to ensure the contractile function of myotubes. In the 2D level, well-arranged murine skeletal myoblasts (C2C12) cells adhered to bilayer sheets through using nanoribbons can promote their differentiation into mature myotubes and help express myogenic genes [40]. Electroconductive nanosubstrates can enhance myogenic differentiation and maturation [41]. However, the 2D culture model might lose the tissue architecture developed during tissue culture, and the number of sheets that can be stacked has an upper limit (i.e., limited thickness) since cells cannot secure nutrients from a distance (e.g., ∼150 *μ* m) which otherwise causes necrosis [42]. When it comes to 3D level, among a variety of scaffold materials, materials with anisotropic architectures, in possession of high similarity in morphology and function to the native tissue, could be an excellent selection to apply to muscle tissue engineering [42]. The well-aligned orientation of muscle tissue, with parallel bundles of muscle fibers, is a guarantee for performing its systolic and diastolic functions. Takahashi et al. [43] has proven that to form an anisotropic myoblast sheets was exactly able to contribute to self-organization behavior and well organize the 3D orientation of myoblasts and myotubes. Chen et al. [44] utilized collagen scaffolds with concave microgrooves to mimic muscle basement membrane and finally found that myoblasts in the engineered muscle tissue highly expressed myosin heavy chain and synthesis of muscle ECM regardless of different groove sizes. To mimic native skeletal muscle tissue, Wang et al. [45] generated hydrogel core-shell scaffolds combining with nanofiber yarns core and successfully induced alignment, elongation, and differentiation of C2C12. Aligned nanofibrous cylinders as scaffolds could be chosen to form aligned, densely populated myotubes, even without a substrate support [46]. Plus, Ku et al. [47] fabricated nanofiber scaffolds with electrical conductivity property and confirmed there is a synergic effect of them in the midst of stimulating muscle cell differentiation. Choi et al. [48] also performed a similar investigation. For volumetric muscle loss (VML) injury, porous collagen-GAG scaffolds implantation could be adopted as a possible good and plausible treatment option to increase muscle hypertrophy and restore functional capacity [49]. In addition to exploiting the chemical or physical attributes of scaffolds, researches of biologic scaffolds for muscle regeneration have recently emerged [50]. Qiu et al. [51] found that mesenchymal stem cells and decellularized ECM scaffold had a synergistic effect on promoting skeletal muscle regeneration. The kind of ECM scaffolds features the ability to modulate macrophage phenotype. However, Dearth et al. [52] have shown that COX1/2 inhibitors such as nonsteroidal anti-inflammatory drugs (NSAIDs), frequently seen in clinical practice and common medications like aspirin, were likely to reduce both collagen content and myogenesis in the defect area, which gives an instruction to pay attention when we apply this technique to patients taking these medications in the future. In the last decade, emerging novel graphene oxide scaffolds have been fabricated to stimulate differentiation and proangiogenic activities of myogenic progenitor cells through mechanical interaction with cells [53]. Besides, Zhao et al. proved that dual bioactive dopamine-incorporated electroactive shape memory elastomers could be applied to soft tissue engineering, especially to skeletal muscle regeneration. There are many other instances of application such as flexible electroactive shape memory copolymers, electroactive ductile polylactide copolymers, and injectable self-healing conductive hydrogels [54]. Accordingly, it can be concluded that synthetic composite materials have displayed unique strengths compared with scaffolds with single structures or materials. When selecting scaffold material, we may make a comprehensive consideration and put the advantages of different materials together as possible as we can to create a composite scaffold in order to better promote cell differentiation in muscle. It is also important to make use of the most appropriate scaffold according to the target tissue. The examples mentioned above are summarized in Table 1.

### 2.5. Environmental Factors Affecting Muscle Differentiation

Muscle stem cells, termed satellite cells, affected by numerous factors, are crucial for skeletal muscle growth and regeneration. The regeneration of skeletal muscle depends on the myogenic differentiation of satellite cells. The most common active promoter of satellite cell proliferation and differentiation in vivo is exercise. One of the most obvious results of exercise is to get function and health state of skeletal muscles improved [55]. The process of myogenic differentiation of stem cells can be divided into two stages. The first stage is cell division, and the second stage is cell differentiation characterized by the expression of certain combinations of myogenic factors [56]. The study of myogenic differentiation of satellite cells has great clinical application potential. For example, this technology may be used to treat VML [9]. When skeletal muscle growth and regeneration are needed, satellite cells will be activated to start myogenic differentiation and then start to proliferate and differentiate into muscle fibers, thus, forming muscle tissue [57]. Pax7 is the guarantee of the function of satellite cells [19]. The growth state of stem cells is closely related to environmental temperature, osmotic pressure, pH value, light, and other factors [58]. For differentiation, the primary importance among them is the mechanical factor because of its role in the cell microenvironment [56, 59]. Moreover, the differentiation of satellite cells is able to be regulated or stimulated by sex hormone [60, 61]. Park et al. found that the differentiation of satellite cells can be activated by electrical stimulation [62]. Common metabolites such as lactic acid, polyamine, and metformin can regulate and stimulate myogenic differentiation [63–66]. In addition, r3h domain containing like (r3hdml) and extractive cells, which are closely related to the cells themselves, can also induce the differentiation of stem cells [67, 68].

### 2.6. Stem Cells for Skeletal Muscle Tissue Engineering

#### 2.6.1. Satellite Cells

Satellite cells, which are also termed muscle stem cells, are located between the basal lamina and sarcolemma of myofibers [69]. The main function of satellite cells is to be responsible for the growth, maintenance, and repair of skeletal muscle after birth, with the ability of self-renewal and differentiation [70]. The paired box transcription factor Pax7 is the specifical gene expressed in satellite cells and is the most important transcription factor to induce satellite cell myogenic differentiation. It is essential for Pax7 to regulate satellite cell expansion and differentiation in both adult and newborn [19, 71]. Pax7 is also absolutely required for skeletal muscle regeneration after acute skeletal muscle injury [72]. H3K4 methyltransferases MLL1 is critical for Pax7 expression and function in vivo. In the absence of MLL1, H3K4me3 at Pax7 and Myf5 promoters are reduced, leading to the decreased expression of Pax7 and Myf5 [18]. It is reported that CD146+ interstitial progenitor cells with no expression of Pax7 have myogenic potential both in vivo and in vitro [57]. MyoD and myf5 are basic regulators determining skeletal muscle lineage in the embryo. They are expressed after muscle injury in satellite cells. The two regulators are essential for muscle regeneration by their stabilizing myogenic identity and giving the capacity for muscle regeneration [73]. CD82 is a novel surface marker for identifying satellite cells isolated from human skeletal muscle. CD82 ensures the expansion and preservation of satellite cells by inhibiting excessive differentiation and it is necessary for satellite cell activation [74–76]. As the adult stem cells of skeletal muscle, satellite cells have been extensively studied and made rapid progress. Prostaglandin E2 (PGE2), which is known as an inflammatory cytokine, can lead to satellite cell expansion by directly targeting satellite cells via the EP4 receptor. Intramuscular delivery of PGE2 can significantly enhance and accelerate the skeletal muscle repair [77]. Notch target genes Hesr1 (Hey1) and Hesr3 (Heyl) are responsible for generating quiescent satellite cells and maintaining the satellite cell numbers [78]. Lysine-specific demethylase 1(Lsd1) can directly regulate key myogenic transcription factor gene to promote muscle regeneration and prevent proadipogenic transcription factor Glis1 differentiating into brown adipocytes [79]. However, although many factors that promote the activation of satellite cells have been researched, they will gradually lose their self-renewal ability as their differentiation. Simultaneously, the main source of satellite cells is skeletal muscle biopsy, and this method will cause great pain to the patient. If a larger amount of satellite cells is needed, it is necessary to biopsy a large number of skeletal muscles, which is almost difficult to achieve clinically. At the same time, the number of satellite cells obtained by the traditional enzymatic dissociation method is small and the purity is low [9]. To solve these limitations, Garcia et al. developed a series of methods for high-grade purification, preservation, and serial transplantation of human satellite cells; these methods provide an accessible system for human satellite cells research and clinical application [80].

#### 2.6.2. Mesenchymal Stem Cells

According to the clarification of The International Society for Cellular Therapy (ISCT), mesenchymal stem cells (MSCs) refer to plastic adherent cells with multidirectional differentiation potential isolated from bone marrow, fat, and other tissues such as umbilical cord blood [81, 82], infrapatellar fat pad [83, 84], and dental tissues [85]. It expresses CD73, CD90, and CD105, but lacking the expression of hematopoietic and endothelial markers CD11b, CD14, CD19, CD34, CD45, CD79a, and HLA-DR. MSC can differentiate into adipocytes, chondrocytes, and osteoblast cell lines in vitro [86, 87]. The two MSCs most commonly used in research are adipose-derived mesenchymal stem cells (ADSCs) and bone marrow-derived stem cells (BMSCs). The bone marrow-derived stem cells are taken from the femur and tibia bone marrow biopsy, which can only obtain a small amount of BMSCs and cause great harm to patients. On the contrary, ADSCs are easier to obtain, faster to grow, and express higher rates of stem cell markers [88]. Thus, the current research about MSCs mainly focuses on ADSCs. Although many studies have shown that MSCs have the effect of promoting muscle regeneration, their mechanism is still unclear. MSCs are multipotent stem cells and have the ability to secrete cytokines and growth factors and have immunoregulatory and proangiogenic abilities [89]. At the same time, it can directly differentiate into muscle tissue in vitro [90]. Under these circumstances, whether MSCs directly differentiate into muscle tissue to replace the damaged muscle tissue or produce paracrine factors to promote muscle regeneration is still controversial. Paracrine factors produced by MSCs such as HGF, bFGF, IGF-1, and VEGF have been confirmed to play key roles in promoting angiogenesis [91]. The latest research found cytokine IL-6 produced by MSCs can stimulate the M2 macrophages to suppress inflammation and regenerate new blood vessels and enhance myogenic differentiation by activating STAT pathway [89, 92]. Mitchell et al. demonstrated ADSCs promote muscle regeneration by its secretome, which contains extracellular vesicle (EV) as well as soluble proteins. EV fraction has anti-inflammatory effects while soluble proteins can reduce the number of senescent cells. Thus, the secretome of ADSCs can promote muscle regeneration both in vivo and in vitro [93]. As for the direct differentiation of MSC into skeletal muscle cells, the current efficiency is still very low. Only 15% of ADSCs can differentiate into skeletal muscle in differentiation medium [94]. Though a number of studies are devoted to promoting its differentiation efficiency, such as culturing cells on scaffolds [95], physical stimulation [96], and chemical stimulation [97]. But the improvement is very limited and not enough to be applied to the clinic. If a paracrine factor that directly promotes differentiation and a method to improve differentiation efficiency can be found, combining the two will greatly promote the application of ADSC in muscle regeneration.

#### 2.6.3. Induced Pluripotent Stem Cells

Induced pluripotent stem cells (iPSCs) were first induced from mouse embryos by introducing specific factors under ES cell culture conditions in 2006 and then induced from adult human fibroblast the next year. Its morphology and growth characteristics are similar to embryonic stem (ES) cells and express ES cell marker genes [98]. Takahashi et al. identified four basic transcription factors, called Yamanaka factors, which must be transformed into starter cells using viral vectors to reprogram the cells into iPSCs: KLF4, c-MYC, OCT4, and SOX2 [98] (see Table 2). Unlike ES cells, iPSCs can derive from almost every adult tissue, and this makes them free of ethical concerns [99]. There are many methods, including transgenic and nontransgenic, to generate a large number of muscle cells from iPSC. Transgenic methods are reliable and can get myogenic progenitors directly. Darabi et al. introduced Pax7 into human ES and iPSC and found that it not only produces a large number of induced myogenic progenitors (iMPCs) with regenerative ability but also contributes to the satellite cell pool and maintains it for a long time after implantation in animals [99]. Culturing iMPC in a 2D environment, it will differentiate into multinucleated myotubes while generating functional skeletal muscle tissues (iSKM bundles) in a 3D hydrogel environment. And iSKM bundles have the biological properties of skeletal muscle such as generating twitch and tetanic contraction. Compared with monolayers in 2D cell culture, iSKM bundles are more similar to native mature muscle. Then, they implanted iSKM bundles into the hindlimb muscle of live mice. Though iSKM bundles are avascular at the first time, ingrown vasculature helped implanted iSKM bundles survival and supported its' function. The 3D culture of IMPCs may be the foundation of PSC-based therapies for muscle regeneration [100]. Nontransgenic methods are easy to do and can be used for research. Shelton et al. developed a protocol for skeletal muscle lineage differentiation from iPSC by using chemically defined media [101]. Wal et al. found that iPSC-derived fluorescence-activated cell sorting-purified myogenic progenitors can expand on a large scale and can develop into striated and contractile myofibers in vitro [102]. To maximum the capacity of unlimited self-renewal and differentiation into any lineage of iPSCs, myogenic progenitors should be produced as pure and easily expandable as possible. CD54, integrin *α*9*β*1, and Syndecan2 (SDC2) are the surface markers of Pax7-induced myogenic progenitors. These markers provide a reliable method to purify iPSC-derived myogenic progenitors for real application [100, 103]. Although many studies have confirmed that iPSC can differentiate into skeletal muscle cells, its disadvantages are also obvious. Immune rejection may be one of the main problems in the clinical application of iPSCs. And due to the inability to precisely control its differentiation direction, iPSC should be thoroughly verified to ensure that they are not carcinogenic [104]. Interestingly, iPSC-derived teratomas show the ability to produce myogenic progenitors. And myogenic progenitors from teratomas can contribute quiescent PAX7+ satellite cells and have functional regenerative capacity [105].

## 3. Conclusions

Skeletal muscle defects and loss of its function due to various causes including congenital defects, injuries, tumors, degenerative pathologies, and metabolic diseases are really common in the clinic. Besides, the risk of certain muscle diseases increases progressively with age. For example, sarcopenia, a progressive and generalised skeletal muscle disorder involving the accelerated loss of muscle mass and function, is common among adults of older age but can also occur earlier in life. The muscle disease burden arises because of their high prevalence all over the world and close relations to short-term and long-term adverse effects. Although skeletal muscle has the ability of regeneration, it depends on the function of satellite cells. After repeated regenerations, the regeneration ability of satellite cells will gradually be impaired. To make muscle regeneration suitable for clinical use, large-scale expansion of satellite cells or differentiation into myogenic lineage from easily obtained stem cells is the main method for skeletal muscle regeneration. Cells differentiated from stem cells cannot become muscle fibers directly. It is the 3D culture environment that makes it possible for muscle cell transforms into skeletal muscle tissue. But there still remains some limitations for application. On the one hand, although many novel methods can produce a lot more cells than before, the differentiation efficiency is still too low. On the other hand, there is too much reliance on transgenic technology and may cause people to worry about safety.

## Figures and Tables

**Table 1:** Classification of different scaffolds.

Material of scaffold/scaffold	Feature	Promoting target	Reference
Graphene oxide scaffolds	Exocrine vascular endothelial growth factor (VEGF) secretion	Myogenic progenitor cells	[53]
Hydrogels based on dextran-graft-tetraaniline and N-carboxyethyl chitosan	Degradable conductive and self-healing	C2C12	[54]
Hydrogel core-shell scaffolds combining with nanofiber yarns core	Mimicking native skeletal muscle tissue	C2C12	[45]
Collagen scaffolds with concave microgrooves	Mimicking muscle basement membrane	Myoblasts	[44]
Uniaxially aligned nanofibrous cylinders	Anisotropy and high surface-to-volume ratio	From myoblasts to myotubes	[46]
Nanofiber scaffolds with electrical conductivity property	Presentation of synergistic effects of different materials	Myoblasts	[47]
Porous collagen-GAG scaffolds	Scaffold implantation	VML injury treatment	[49]
Mesenchymal stem cells and extracellular matrix scaffolds	Functioning via promoting macrophage polarization toward the M2 phenotype and suppress macrophage polarization toward the M1 phenotype	Macrophage	[51]

**Table 2:** Stem cells in myogenic differentiation.

Stem cell types	Stem cell sources	Markers	Advantages
Satellite cells	Muscle biopsy	HEYL, KLF4, MYOD, PAX7, Myf5, CD82	Direct precursor of skeletal muscle
Mesenchymal stem cells	Bone marrow biopsy (BMSC), adipose tissue (ADSC), and other mesenchymal tissues	CD73, CD90, CD105 and lacking CD11b, CD14, CD19, CD34, CD45, CD79a	Easy to obtain and low carcinogenic risk
Induced pluripotent stem cells	Almost every adult tissue	KLF4, c-MYC, OCT4, SOX2	Pluripotent differentiation potential and high differentiation efficiency
